# Success of transition to adult care in patients with pediatric‐onset chronic liver disease

**DOI:** 10.1002/jpn3.70436

**Published:** 2026-04-20

**Authors:** Sarah Mongbo, Valérie Canva, Sébastien Dharancy, Guillaume Lassailly, Alexandre Louvet, Philippe Mathurin, Nassima Ramdane, Frédéric Gottrand, Madeleine Aumar

**Affiliations:** ^1^ Department of Paediatric Gastroenterology, Hepatology and Nutrition Inserm U1286 Infinite, CHU Lille Université de Lille Lille France; ^2^ CHU Lille, Unit of Gastroenterology and Hepatology, Inserm U1286 Infinite Université de Lille Lille France; ^3^ Department of Biostatistics CHU Lille Lille France

**Keywords:** children, evolution, failure, hepatopathy, risk factor

## Abstract

**Objectives:**

Previous studies on chronic pediatric‐onset conditions have highlighted the risks of loss to follow‐up, disease progression, or therapeutic nonadherence during transition. However, very few studies have focused on liver diseases. We aimed to evaluate the rate of successful transition in a cohort of patients with chronic liver disease, and assess its evolution over time and factors associated with transition failure.

**Methods:**

We conducted an observational, retrospective, monocentric study at our tertiary hospital. Ninety‐three patients attending the pediatric hepatology clinic who were transferred to the adult‐oriented hepatology clinic between 1997 and 2019 with at least 2 years of follow‐up after transfer were included. Transition failure was defined as death, a history of poor medical office visit attendance, or complications due to therapeutic nonadherence within 2 years after transfer.

**Results:**

The prevalence of transition failure was 24%. Of this, poor attendance was 68% and complication was 32%, no deaths. The failure rate of transition decreased by almost 80% over time. A history of therapeutic nonadherence (odds ratio (OR) = 3.49; confidence interval (CI) 1.17–10.43, *p* = 0.03) and nonattendance to at least one pediatric consultation (OR = 2.76; CI 1.06–7.15, *p* = 0.04) in the year before transfer were the only risk factors for transition failure. Significantly more patients in the success group than in the failure group had met an adult‐oriented hepatologist before transfer (31% vs. 9.1%, respectively, *p* = 0.04).

**Conclusions:**

Transition was successful in more than three‐quarters of our cohort and even improved over time.

## Abstract (highlights)

**What is Known:**

• In other chronic medical conditions, research has shown the transition to be an at‐risk period for the impairment of adolescent and young adult health status.

**What is New:**

• We found that 24% of patients with pediatric‐onset chronic liver disease had a failed transition to adult care.
• We identified that those who struggled with adherence to medical management in the years leading up to the transition—were more likely to experience this failure.

## Graphical abstract

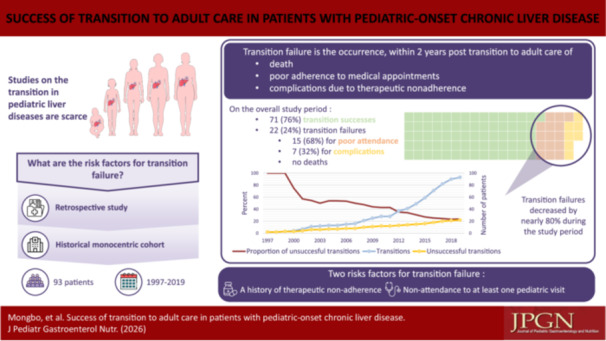

## 1 INTRODUCTION

In other chronic conditions, transition is a high‐risk period for health deterioration in adolescents and young adults, sometimes resulting in premature death.^1^, ^2^, ^3^, ^4^, ^5^ Over half of patients either never engage adult‐oriented care or are lost to follow‐up within 24 months^6^, ^7^ with therapeutic nonadherence being a major concern. In pediatric liver transplant patients, Annunziato et al. showed a significant increase in nonadherence, exemplified by an increase in residual tacrolimus level variability (indicative of irregular use of tacrolimus), from pre‐transition to 2 years post‐transition.^8^ Two studies reported a 25%–30% rise in mortality, with one‐third of deaths occurring within the first post‐transition year,^9^, ^10^ others oted a 25% decline in consultation attendance during the 2 years following transition.^9^ Data on transition in pediatric liver diseases remain limited, and risk factors in this population are poorly understood.

The primary aim of our study was to assess the prevalence of transition failure in a cohort of patients with pediatric chronic hepatic liver disease. Secondary objectives were to assess the factors associated with transition failure and its evolution over time.

## 2 METHODS

### 2.1 Ethics statement

This study was approved by the *Groupe Francophone d'Hépatologie‐Gastroentérologie et Nutrition Pédiatriques* (Francophone Group of Pediatric Hepatology, Gastroenterology, and Nutrition) ethics committee. It was also declared to the *Commission nationale de l'informatique et des libertés* (National Commission on Informatics and Liberty) with the identification number 1309. Information and non‐opposition letters were sent to all eligible patients for the study. None of them expressed refusal to participate.

### 2.2 Study design

We conducted an observational, retrospective, monocentric study at our tertiary hospital. We included every pediatric patient with chronic liver disease followed in the University Hospital of Lille for at least 1 year before transfer, who had a minimum of two pediatric consultations between 1997 and 2019, and who were transfered to the adult‐oriented hepatology clinic at the center between January 1997 and October 2019. Patients transfered to another medical center or whose transition was made by another pediatric specialist than a hepatologist were excluded. The follow‐up period after the transfer was at least 2 years.

Data were retrospectively extracted from the patients' files. We collected patients' demographics, other chronic conditions, information about the liver disease (type, date of diagnosis, evolution of complicated liver disease, frequency of consultations, and pediatric care duration), therapeutic adherence, appointment attendance, disease stability, duration of the transition process, date of transfer, and contact with adult‐oriented providers before transfer. Adult hepatologist experience was also recorded and stratified by the number of transitioned patients each hepatologist was following: less than 10; between 10 and 40; and more than 40. INSEE death registers (*Institut national de la statistique et des études économiques*, The National Institute of Statistics and Economic Studies) were consulted to ensure that there were no unknown deaths among the patients lost to follow‐up.

### 2.3 Definitions

Complicated liver disease was defined as a liver disease with hepatic insufficiency, cirrhosis, portal hypertension, relapse, or the requirement for liver transplantation, at any time before transfer. Liver disease was characterized as stable before transfer if none of the following occurred within the year before transfer: complication, therapeutic escalation, or relapse. Complications were defined as the occurrence or worsening of cirrhosis, hepatic insufficiency, or portal hypertension, or as a rise in transaminases, gamma‐glutamyl transferase (≥50% from baseline), or bilirubin (≥35 µmol/L from baseline). Cirrhosis was diagnosed histologically or by clinical, imaging, and elastography findings (liver stiffness > 20 kPa), with worsening indicated by an elastography increase ≥ 5 kPa. Hepatic insufficiency was defined by serum albumin < 35 g/L and prothrombin time or factor V < 50%, with worsening marked by a ≥ 50% decrease in prothrombin time from baseline. Portal hypertension was identified by portosystemic collaterals or varices on endoscopy/imaging, with worsening defined by new varices or a ≥1‐grade increase in esophageal varices. Therapeutic escalation was defined by an increase in dosage, an addition or a change of therapy to improve disease control. Relapse was defined by the reappearance of biological or histological markers of disease activity that were previously absent after initial treatment in patients with viral or autoimmune liver disease. Transition failure was defined by the occurrence of death of any origin, poor medical office visit attendance, or complication (as defined above) due to therapeutic nonadherence within 2 years after transfer. Poor medical office visit attendance was defined as nonattendance at the first or second office visit at the adult‐oriented center within 2 years after transfer, if a second consultation was requested by the hepatologist in his consultation letter. Therapeutic nonadherence was defined its mention by the pediatrician in the patient's file whether or not it was based on biological measurements of medication when applicable. Therapeutic nonadherence prior to transition was considered as a factor for transition failure, while nonadherence to treatment after transfer was an outcome.

The evidence of transfer from the pediatric unit to the adult center consisted of a letter of referral from the pediatric hepatologist or a confirmation letter from the adult‐oriented hepatologist to the pediatric hepatologist of their agreement to follow the patient.

The timing of transfer was defined by the date of the last visit to the pediatric hepatologist. The duration of the transition process was the time between when the transfer was first mentioned in a pediatrician's letter and the date of the last visit to the pediatric hepatologist.

### 2.4 Transition protocol

Despite limited resources and the absence of a dedicated transition coordinator, transition was individualized for each adolescent and family based on disease and personal needs, after holding individual discussions with the adolescent and, separately, with their family. From age 15—and at least 1 year before transfer—a flexible, tailored plan was established, involving the adolescent and family. Support included adapted informational materials (e.g., brochures, en.filfoie.com), and social worker access as needed. Readiness and acceptance were reassessed during subsequent consultations. The adult hepatologist's identity and the transition timeline (last pediatric visit and first adult appointment within 6 months) were clearly communicated. Adult hepatologists received comprehensive medical and socio‐familial documentation. In complex cases (clinical, psychosocial, or compliance concerns), the adult hepatologist attended the final pediatric visit and/or the pediatrician attended the first adult visit. Hospitalization for acute decompensation or transplantation was avoided whenever possible. Non‐attendance triggered a “no‐show” letter and notification to the pediatric hepatologist.

### 2.5 Statistical analysis

Categorical variables are expressed as numbers (percentage). Continuous variables are expressed as means (standard deviation [SD]) in the case of normal distribution or medians (interquartile range [IR]) otherwise. Normality of distribution is assessed using histograms and the Shapiro–Wilk test. Categorical variables are compared according to the transfer failure, then according to the adult contact groups, using chi‐square tests (or Fisher's exact tests when the expected cell frequency was < 5), and using the Mann–Whitney *U* test for non‐Gaussian continuous variables and the Student t‐test for Gaussian continuous variables. For risk factors for transfer failure, odds ratios (OR) are also calculated to measure the effect size with their 95% confidence interval (CI) by a logistic regression model. Statistical testing is done at the two‐tailed *α* level of 0.05. Data are analyzed using SAS software package, release 9.4 (SAS Institute, Cary, NC, USA). Due to the small number of patients in the transition failure group, multivariate analysis was not feasible because of insufficient statistical power.

## 3 RESULTS

**Figure 1:**
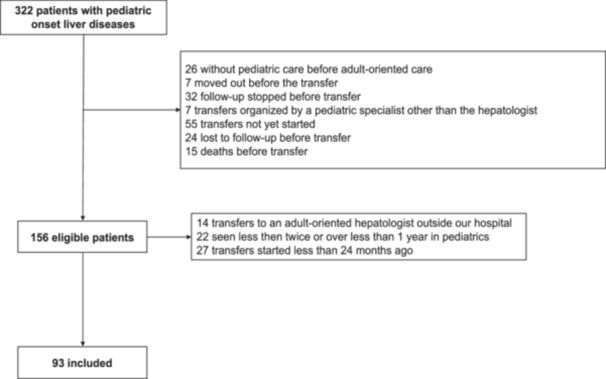
Flow chart.

We included 93 out of the 156 eligible patients (60%) in the study. The study flow chart is presented in Figure 1.

### 3.1 Population

Among the included population, 62.4% were female and 45.2% had another associated chronic condition. The median (IR) age of onset of the disease was 5 years (0–12), with a median (IR) duration of pediatric follow‐up of 12 years (4–17). The three most common hepatic conditions were autoimmune hepatitis (18.3%), biliary atresia (14.0%), and chronic hepatitis B (11.8%).

Eighty‐two percent of patients were asymptomatic at transfer, and 41.9% had a complicated liver disease, including 17.2% with a history of liver transplantation. Eleven percent (*n* = 10) of the patients had one office visit nonattendance in the year prior to transfer, and 3.2% had two (*n* = 3). Seventy‐one patients had a successful transition (76%). There was therapeutic nonadherence the year before transfer for 19.3% of patients, with a significant difference between the two groups (36.4% in the transition failure group vs. 14.1% in the successful transition group, *p* = 0.03).

The median age (IR) at transfer in the population was 17 years (17–18) after a mean preparation time of 6 months (0.5 ± 0.7). Twenty‐six percent of patients (*n* = 24) had contact with an adult hepatologist prior to transfer with a significant difference between the two groups (9.1% in the transition failure group vs. 31% in the successful transition group, *p* = 0.04). Nineteen patients met an adult hepatologist during a follow‐up transient elastography before transfer. One patient had visited the adult hospital unit prior to transfer, one had an alternate consultation, and one had a joint consultation. Three patients met an adult hepatologist as part of a liver transplant plan during an emergency hospitalization in pediatrics. The characteristics of the population according to the transition outcome are presented in Table 1.

**Table 1:** Population characteristics depending on transition outcome.

	All	Successful transition	Transition failure	*p*‐value	Missing data
	*n* = 93	*n* = 71	*n* = 22		
Female *n* (%)	58 (62.4)	43 (60.6)	15 (68.2)	0.52	
BMI med (IR)	22.0 (20.0–24.0)	22 (20.0–24.0)	21.0 (20.0–23.5)	0.63	2
Associated chronic diseases *n* (%)	42 (45.2)	33 (46.5)	9 (40.9)	0.64	
Psychiatric	7 (7.5)	4 (5.6)	3 (13.6)	*NA*	
Neurodevelopmental	8 (8.6)	7 (9.9)	1 (4.5)	0.67	
Others	35 (37.6)	29 (40.8)	6 (27.3)	0.25	
Other specialized medical care *n (%)*	35 (37.6)	28 (39.4)	7 (31.8)	0.52	
Type of hepatopathy *n* (%)				0.19	
Biliary disease				0.80	
Biliary atresia	13 (14.0)	11 (15.5)	2 (9.1)		
Alagille syndrome	7 (7.5)	5 (7.0)	2 (9.1)		
Congenital liver fibrosis	2 (2.2)	1 (1.4)	1 (4.5)		
Common bile duct cyst	1 (1.1)	1 (1.4)	0 (0.0)		
Genetic, metabolic, toxic, or storage disease				1.00	
Wilson's disease	7 (7.5)	3 (4.2)	4 (18.2)		
Ornithine transcarbamylase deficiency	2 (2.2)	2 (2.8)	0 (0.0)		
Crigler Najar syndrome	1 (1.1)	1 (1.4)	0 (0.0)		
Lysosomal acid lipase deficiency	1 (1.1)	1 (1.4)	0 (0.0)		
Parenteral induced cirrhosis	1 (1.1)	1 (1.4)	0 (0.0)		
MAFLD	1 (1.1)	1 (1.4)	0 (0.0)		
Congenital cholestasis	6 (6.5)	5 (7.0)	1 (4.5)		
Immune, vascular, tumoral, and undetermined disease				0.15	
Autoimmune hepatitis	17 (18.3)	12 (16.9)	5 (22.7)		
Primary sclerosing cholangitis	4 (4.3)	4 (5.6)	0 (0.0)		
Overlap syndrome	4 (4.3)	4 (5.6)	0 (0.0)		
Portal cavernoma	2 (2.2)	2 (2.8)	0 (0.0)		
Fanconi disease	1 (1.1)	1 (1.4)	0 (0.0)		
Abernathy syndrome	1 (1.1)	1 (1.4)	0 (0.0)		
Cardiac liver	1 (1.1)	1 (1.4)	0 (0.0)		
Hepatoportal sclerosis	1 (1.1)	1 (1.4)	0 (0.0)		
Hepatoblastoma	1 (1.1)	1 (1.4)	0 (0.0)		
Nodular and focal hyperplasia	1 (1.1)	1 (1.4)	0 (0.0)		
Undetermined cirrhosis	2 (2.2)	2 (2.8)	0 (0.0)		
Viral disease				0.053	
Hepatitis B	11 (11.8)	6 (8.5)	5 (22.7)		
Hepatitis C	4 (4.3)	3 (4.2)	1 (4.5)		
Hepatitis A^a^	1 (1.1)	0 (0.0)	1 (4.5)		
Age at diagnostic med (IR)	5.0 (0.0–12.0)	4.0 (0.0–12.0)	10.5 (1.0–13.0)	0.14	
Symptomatic patient *n* (%)	17 (18.3)	14 (19.7)	3 (13.6)	0.75	
Complicated liver disease *n* (%)	39 (41.9)	31 (43.7)	8 (36.4)	0.54	
Liver transplant	16 (17.2)	13 (18.3)	3 (13.6)	0.75	
Retransplantation	3 (3.2)	3 (4.2)	0 (0.0)	*NA*	
Portal hypertension	27 (29.0)	21 (29.6)	6 (27.3)	0.83	
Cirrhosis	17 (18.3)	13 (18.3)	4 (18.2)	1.00	
Hepatic insufficiency	3 (3.2)	2 (2.8)	1 (4.5)	*NA*	
Relapse	4 (4.3)	2 (2.8)	2 (9.1)	*NA*	
Mean time between pediatric consultations (in months) med (IR) ± SD	12.0 (6.0–12.0)	12.0 (6.0–12.0)	9.0 (6.0–12.0)	0.72	1
Follow‐up time before transfer (in years) med (IR)	12 (4.0–17)	12.0 (4.0–18.0)	6.5 (4.0–17.0)	0.18	
Unstable disease before transfer *n* (%)	24 (25.8)	19 (26.8)	5 (22.7)	0.71	
Office visit nonattendance *n* (%)	13 (14)	7 (9.9)	6 (27.3)	0.07	
Once	10 (10.7)	6 (8.4)	4 (18.2)	*NA*	
Twice	3 (3.2)	1 (1.4)	2 (9.1)	*NA*	
Therapeutic nonadherence before transfer *n* (%)	18 (19.3)	10 (14.1)	8 (36.4)	**0.03**	
Distance between residence and hospital *n* (%)				**0.03**	
>70 km	23 (24.7)	17 (23.9)	6 (27.3)		
Between 45 and 70 km	21 (22.6)	20 (28.2)	1 (4.5)		
Between 17 and 45 km	25 (26.9)	20 (28.2)	5 (22.7)		
<17 km	24 (24.8)	14 (19.7)	10 (45.4)		
Age at transfer med (IR)	17 (17–18)	17 (17–18)	17.5 (17–18)	0.14	
Time of transfer in regard to high school graduation *n* (%)				0.74	6
Before	30 (34.5)	22 (33.8)	8 (36.4)		
Year of graduation	35 (40.2)	27 (41.5)	8 (36.4)		
After	15 (17.2)	10 (15.4)	5 (22.7)		
Not concerned by high school graduation	7 (8.0)	6 (9.2)	1 (4.5)	*NA*	
Transition preparation time (in years) med (IR)	0 (0–1)	0 (0–1)	0 (0–1)	0.67	
Contact with adult‐oriented unit before transfer *n* (%)	24 (25.8)	22 (31)	2 (9.1)	**0.04**	
Adult‐oriented hepatologist experience *n* (%)				0.14	
<10 transitions	15 (16.1)	9 (12.7)	6 (27.3)		
Between 10 and 40 transitions	18 (19.3)	16 (22.5)	2 (9.1)		
>40 transitions	60 (64.5)	46 (64.8)	14 (63.6)		

*Note*: Overlap syndrome, association of autoimmune hepatitis and primary sclerosing cholangitis. Bold values indicate statistically significant.

Abbreviations: BMI, body mass index; IR, interquartile range; MAFLD, metabolic dysfunction‐associated steatotic liver disease; NA, not applicable; SD, standard deviation.

^a^ Liver transplanted patient.

The number of patients who had met an adult hepatologist before transfer over time is shown in Supporting Information: Figure S1. A visual cut‐off at 2012 is apparent from the graph which is statistically confirmed as more patients met an adult hepatologist before transfer after 2012 than before (32.3% vs. 10.7% *p* = 0.03).

Population characteristic depending on transition timing (before or after 2012) are shown in Supporting Information: Figure S2. The only significant difference between the two groups was BMI, with a mean BMI of 22.4 before 2012 and 23.0 after 2012 (*p* = 0.048).

### 3.2 Success of the transition

Twenty‐two patients (23.7%) had a transition failure. Fifteen patients (68.2%) had poor medical office visit attendance. Seven of them (31.8%) were then lost to follow‐up within an average period of 2.4 ± 4.8 months after transfer. The other seven failures (31.8%) had a complication related to therapeutic nonadherence. There were no deaths declared in the cohort. In the successful transition group, one patient was finally lost to follow‐up after two office visits with the adult‐oriented hepatologist. Patient outcomes after transfer are shown in Supporting Information: Figure S3.

### 3.3 Factors associated with transition failure

Univariate analysis of potential factors associated with a transition failure is presented in Table 2. A history of treatment nonadherence (OR = 3.49, 95%CI 1.17–10.43) and nonattendance at the office visit before transfer (OR = 2.76, 95%CI 1.06–7.15) were the only factors significantly associated with a transition failure.

**Table 2:** Factors associated with unsuccessful transition.

	Successful transition	Transition failure	*p*‐value	OR	95%CI
	*n* = 71	*n* = 22			
Female *n* (%)	43 (60.6)	15 (68.2)	0.52	1.39	0.50–3.85
Comorbidities *n* (%)	33 (46.5)	9 (40.9)	0.65	0.80	0.30–2.10
Neurodevelopmental	7 (9.9)	1 (4.5)	0.45	0.43	0.05–3.75
Psychiatric	4 (5.6)	3 (13.6)	0.23	2.64	0.54–12.86
Other specialized medical care *n* (%)	28 (39.44)	7 (31.82)	0.52	0.72	0.26–1.98
Age at diagnosis *m* ± SD	5.5 ± 5.7	7.8 ± 5.7	0.11	1.07	0.98–1.17
Pathology groups *n* (%)			0.2		
Bile duct disease	18 (25.3)	5 (22.7)			
Genetic, metabolic, toxic, or storage disease	16 (22.5)	5 (22.7)		1.12	0.27–4.61
Dysimmunitary, vascular, tumoral, and undetermined disease	28 (30.1)	5 (22.7)		0.64	0.16–2.54
Viral hepatitis (HAV, HBV, and HCV)	9 (12.7)	7 (31.8)		2.80	0.69–11.34
Symptomatic patient *n* (%)	14 (19.7)	3 (13.6)	0.52	0.64	0.17–2.48
Complicated liver disease *n* (%)	31 (43.7)	8 (36.4)	0.54	0.74	0.27–1.98
Liver transplant	13 (18.3)	3 (13.6)	0.61	0.7	0.18–2.74
Mean time between pediatric consultations (in months) *m* ± SD	8.89 ± 3.34	9.09 ± 4.89	0.82	1.01	0.89–1.15
Follow‐up time before transfer (in years) *m* ± SD	11.6 ± 6.1	9.4 ± 6.1	0.14	0.94	0.87–1.02
Unstable disease before transfer *n* (%)	19 (26.8)	5 (22.7)	0.66	0.71	0.26–2.49
Pediatrics office visit nonattendance *n* (%)	7 (9.9)	6 (27.3)	**0.04**	**2.76**	**1.06–7.15**
Therapeutic nonadherence before transfer *n* (%)	10 (14.1)	8 (36.4)	**0.03**	**3.49**	**1.16–10.43**
Transfer before 18 years old *n* (%)	40 (56.3)	11 (50.0)	0.6	0.77	0.30–2.02
Time of transfer in regard to high school graduation *n* (%)			0.74		
Before	22 (33.85)	8 (36.4)			
Year of the graduation	27 (41.5)	8 (36.4)		0.81	0.26–2.52
After	10 (15.4)	5 (22.7)		1.37	0.36–5.27
Transfer preparation time (in years) *m* ± SD	0.52 ± 0.61	0.59 ± 0.96	0.40	0.66	0.25‐1.76
Contact with adult‐oriented unit before transfer *n* (%)	22 (31)	2 (9.1)	0.056	0.22	0.05–1.04
Adult‐oriented hepatologist experience *n* (%)			0.17		
<10 transitions	9 (12.7)	6 (27.3)		Reference	
Between 10 and 40 transitions	16 (22.5)	2 (9.1)		0.19	0.03–1.13
>40 transitions	46 (64.8)	14 (13.6)		0.46	0.14–1.51
Distance between residence and adult‐care hospital *n* (%)			0.033		
>70 km	17 (23.9)	6 (27.3)		Reference	
Between 45 and 70 km	20 (28.2)	1 (4.5)		2.02	0.59–6.96
Between 17 and 45 km	20 (28.2)	5 (22.7)		0.71	0.18–2.74
<17 km	14 (19.7)	10 (45.4)		0.14	0.01–1.29

*Note*: Bold values indicate statistically significant.

Abbreviations: CI, confidence interval; HAV, hepatitis A virus; HBV, hepatitis B virus; HCV, hepatitis C virus; OR, odds ratio; SD, standard deviation.

None of the factors related to the transition process were associated with the success of the transition.

### 3.4 Evolution of the success of transition with time

**Figure 2:**
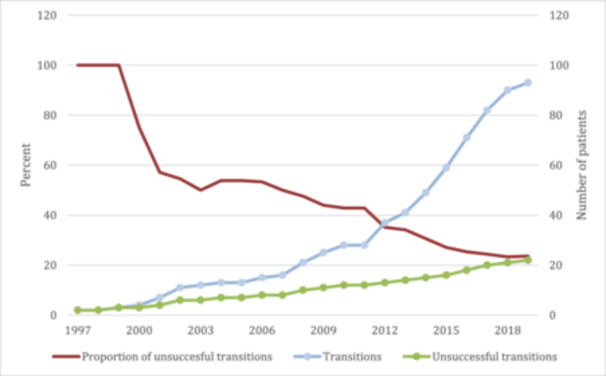
Evolution of the cumulative number of all transitions and transition failures.

The evolution of the success of transitions over time is shown in Figure 2.

We observed were more successful transitions after 2012 than before (84.6% vs. 57.1% respectively, *p* = 0.004).

## 4 DISCUSSION

The failure of transition in pediatric‐onset chronic liver disease is frequent and affected almost a quarter of our cohort. However, this prevalence is lower than those reported in other pediatric‐onset chronic conditions (i.e., diabetes, congenital heart disease, inflammatory bowel disease [IBD]), which vary from 30% to 60%.^3^, ^6^, ^7^, ^11^, ^12^, ^13^, ^14^ These differences may be explained by differences in the definition of “failure”; to date, there is no consensus.^15^, ^16^ Some teams use disease‐specific outcomes, for example, hemoglobin A1C in diabetes or tacrolimus levels in transplant patients.^16^ Our definition is applicable to every pediatric‐onset liver disease. Some teams analyze all disease‐related hospitalizations.^15^, ^16^ We have chosen only to take into account complications related to therapeutic nonadherence. We considered, in other cases, that it was the natural evolution of the disease. Unlike the majority of studies,^7^, ^12^, ^13^ we have defined transition failure as a failure to attend a second office visit over the 24 months following the transfer (instead of the first visit). Indeed, the first contact with the adult‐oriented team does not always lead to adherence to follow‐up over time.^9^ Gleeson et al. found that attendance at the first two appointments is “a reasonable marker of ongoing engagement in adult care.”^17^

Currently, it is recommended to set up the transition during a period of disease stability and to limit modifications to therapy immediately after transfer.^18^, ^19^ Some teams consider treatment escalation after transfer as evidence of an unstable pathology and inappropriate transfer timing^11^ In our cohort, 25% of patients had treatment intensification after transfer with no significant difference between the success group and the failure group. Indeed, we found that therapeutic modification does not systematically indicate a worsening of the disease. It can, for example, be only related to the possibility of accessing adult‐only medications, as was the case for patients with hepatitis C virus in our study. The effect of transition on disease stability itself could not be assessed in this study.

A history of therapeutic nonadherence in the year before the transfer and a history of nonattendance at medical office visits are the only risk factors for a transition failure in our population. Therapeutic nonadherence has also been reported as a risk factor for suboptimal transition in pediatric IBD.^11^ To our knowledge, a history of nonattendance at medical office visits has never before been considered a risk factor. These risk factors reflect a preexisting poor adherence to medical care that is more likely to persist after the transfer when the patient is confronted by a team they do not know or trust. Two previous adult studies have also reported therapeutic nonadherence to be one of the greatest barriers to optimal care in liver transplantation.^20^, ^21^

The existence of symptoms at the time of transfer was not associated with a transition failure in our study, even if the association between low‐activity disease and a transition failure has been previously described in other chronic conditions.^12^, ^22^, ^23^ We chose to test the correlation of the two most frequent symptoms of chronic liver disease (pruritus and jaundice) with transition outcome, but we had no quality‐of‐life data for our patients. The same reported symptoms may have a different impact on everyday life, depending on the individual. Patients complaining about their disease may have better therapeutic adherence compared with patients who feel they are not ill. Nevertheless, previous studies have shown that symptoms (pruritus, fatigue, and abdominal pain among others) are independently associated with a reduced quality of life in children and adolescents with autoimmune hepatitis, liver transplant at pediatric age, or nonalcoholic fatty liver disease.^24^, ^25^, ^26^, ^27^, ^28^, ^29^ Analysis of the impact of these symptoms on transition is therefore relevant even in the absence of data on quality of life.

Over 22 years, transition failures decreased by 80%, inversely correlated with a marked rise in transitioned adolescents. Notably, a single pediatric hepatologist managed all included patients, whereas adult hepatologists' experience did not influence transition success. These results mays underscore the pivotal role of pediatricians' transition process expertise and accumulated experience. While transition importance is now widely recognized and supported by scientific society guidelines, awareness and practice have evolved significantly over the past two decades. Increased patient caseloads have further enhanced practical experience. To our knowledge, this is the first study demonstrating the impact of pediatric specialist experience on transition outcomes.

Study limitations include its retrospective design, with potential information bias due to incomplete data—particularly regarding treatment adherence—though no eligible patient (*n* = 156) was excluded for missing transition data. Population heterogeneity in underlying liver diseases necessitated broad definitions, potentially biasing intergroup comparisons. Given the rarity of pediatric‐onset chronic liver diseases, restricting analysis to a single entity was infeasible; however, as underlying pathology did not affect outcomes, identified risk factors for transition failure may generalize across such diseases.

Another limitation of our work is the small size of the transition failure group, which may lead to a lack of power to show significant differences, despite a relatively large population of 93 patients. Pediatric‐onset hepatopathies are rare diseases, and fortunately, transitions are successful most of the time. The relatively small number of patients with transition failure probably explains why the distance between the patient's place of residence and Lille University Hospital was not significant as a risk factor, whereas there was a significant difference between the failure and non‐failure groups. Distance from the hospital has previously been shown by others to be associated with transition failure.^12^, ^22^ Similarly, psychiatric comorbidities and preliminary referring to an adult‐oriented team before transfer did not reach significance in the association with transition failure, as has previously been shown.^9^, ^11^ Negative beliefs about adult care are a common patient barrier,^30^ and meeting the adult‐oriented team prior to transfer increased feelings of security and confidence and facilitated the development of patient relationships with their new providers.^31^ In our cohort, 92% of patients who had preliminary contact with an adult hepatologist had a successful transition. Most patients who had an encounter with an adult hepatologist did so after 2012, and the transition success rate is better after 2012 than before. No differences were observed between patients who transitioned before 2012 and those who transitioned after 2012 regarding sex, age at diagnosis, follow‐up duration, pathology group, comorbidities, or history of missed appointments. Similarly, there were no differences in complications, disease instability, or therapeutic nonadherence before transition. The only significant difference identified was in BMI, though both groups had mean values within the normal range. Despite the fact that this is not an ideal transition and does not meet the recommendation specifications, we observe that a contact with an adult hepatologist, even if only during a transient elastography, seems to be a protective factor. This reinforces the fact that adult hepatologists need to be more involved in the transition process to get closer to these guidelines, and to improve transition outcome.

Our study also has strengths. Due to the organization of care in our geographical area of *Hauts‐de‐France*, most children who were transitioned to adult‐oriented hepatologists in the period of interest have been included in our study. Indeed, pediatric liver diseases are predominantly rare diseases whose management requires highly specialized pediatric and adult‐oriented care. Lille University Hospital is the only pediatric and adult‐oriented reference center in the *Hauts‐de‐France* region for these pathologies. Only 9% of the patients in our eligible population was transitioned to other adult‐oriented hepatologists. This study is an exhaustive population‐based cohort study and a reflection of the population of patients with pediatric‐onset liver disease in *our region*. It is also a historical cohort that reflects more than 20 years of our practice. Its monocentric design by a single pediatrician highlights the impact of the pediatrician in the transition. To our knowledge, this study is unique in its investigation of the factors associated with transition failure in patients with pediatric‐onset chronic liver disease. Previous studies have shown that a structured transition program is linked to a better outcome, with stronger therapeutic adherence, less nonattendance at adult‐oriented care, fewer hospitalizations, and even lower mortality after transition.^32^, ^33^, ^34^

## 5 CONCLUSION

Transition was successful in more than three‐quarters of our cohort and even improved over time with the experience of the process. The pediatric‐to‐adult care transition represents a critical phase for adolescents and young adults with chronic disease requiring anticipation and preparation. Our study suggests clinicians should pay special attention to patients with a history of therapeutic nonadhrence or poor attendance at consultations before transition who may need stronger support and follow‐up.

## CONFLICT OF INTEREST STATEMENT

Sébastien Dharancy reports receiving lecture fees from Chiesi, Astellas, Roche, Ipsen and Abbvie. Valérie Canva reports receiving lecture fees from Abbvie and Gilead. Frédéric Gottrand is a member of the advisory board of Mirum and Ipsen.

## Supporting information

Supplementary Figure S1 Contact with an adult hepatologist before transfer over time.

Supplementary Figure S2 Population characteristics depending on transition timing before or after 2012.

Supplementary Figure S3 Patient outcomes after transfer.
