# Supplementary material for: Success of transition to adult care in patients with pediatric‐onset chronic liver disease
Source: J Pediatr Gastroenterol Nutr. 2026 Apr 20;83(1):145–54. doi: 10.1002/jpn3.70436 (PMC13342795; doi:10.1002/jpn3.70436)
Supplement: Supplementary file 2 — Supplementary Figure S2 Population characteristics depending on transition timing before or after 2012. [file JPN3-83-145-s002.docx]

|  | **Before 2012** | **After 2012** | ***p*** |
| --- | --- | --- | --- |
|  | **n = 28** | **n = 65** |  |
|  |  |  |  |
| Female *n (%)* | 18 (64.3) | 40 (61.5) | 0.80 |
| BMI *m ± SD* | 22.4 ±3.6 | 23.0 ±4.4 | **0.048** |
| Comorbidities *n (%)* | 16 (57.1) | 26 (40.0) | 0.13 |
|  |  |  |  |
| Pathology groups *n (%)* |  |  | 0.09 |
| Bile duct disease | 6 (21.4) | 17 (26.2) |  |
| Genetic, metabolic, toxic or storage disease | 10 (35.7) | 9 (13.8) |  |
| Dysimmunitary, vascular, tumoral and undetermined disease | 7 (25.0) | 28 (43.1) |  |
| Viral diseases: | 5 (17.9) | 11 (16.9) |  |
| Age at diagnostic *m ± SD* | 5,8 ± 5,4 | 6.1 ± 5.9 | 0.90 |
| Symptomatic patient *n (%)* | 5 (17.9) | 12 (18.5) | 0.94 |
| Complicated liver disease *n (%)* | 13 (46.4)) | 26 (40.0) | 0.56 |
| Follow-up time before transition (in years) *m ± SD* | 11.3 ± 6.4 | 11.0 ± 5.9 | 0.90 |
| Unstable disease before transition *n (%)* | 7 (25.0) | 17 (26.2) | 0.91 |
| Office visit nonattendance *n (%)* | 7 (25.0) | 6 (9.2) | 0.056 |
| Therapeutic non-compliance before transition *n (%)* | 7 (25.0) | 11 (16.9) | 0.37 |
|  |  |  |  |
| Distance between residence and Hospital *n (%)* | 54.0 ± 36.0 | 74.6 ± 245.1 | 0.28 |
| BMI: body mass index |  |  |  |
