# Supplementary material for: Success of transition to adult care in patients with pediatric‐onset chronic liver disease
Source: J Pediatr Gastroenterol Nutr. 2026 Apr 20;83(1):145–54. doi: 10.1002/jpn3.70436 (PMC13342795; doi:10.1002/jpn3.70436)
Supplement: Supplementary file 3 — Supplementary Figure S3 Patient outcomes after transfer. [file JPN3-83-145-s001.docx]

|  | **All** | **Successful** | **Transition** | ***p*** |
| --- | --- | --- | --- | --- |
|  |  | **transition** | **failure** |  |
|  | **n = 93** | **n = 71** | **n = 22** |  |
|  |  |  |  |  |
| Hospitalization after transfer *n* *(%)* | 6 (6.45) | 3 (4.2) | 3 (13.6) | *NA* |
| Hospitalization after transfer due to therapeutic nonadherence | 3 (3.2) | 0 (0.0) | 3 (13.6) | *NA* |
| Complication after transfer *n* *(%)* | 13 (14) | 7 (9.9) | 6 (27.3) | 0.07 |
| Complication after transfer due to therapeutic nonadherence | 5 (5.38) | 0 (0.0) | 5 (22.7) | *NA* |
| Therapeutic escalation after transfer *n* *(%)* | 23 (24.7) | 18 (25.3) | 5 (22.7) | 0.83 |
| Office visit nonattendance at the adult-oriented center *n (%)* | 29 (31.2) | 22 (31.0) | 7 (31.8) | 0.058 |
| Once | 18 (19.3) | 14 (19.7) | 4 (18.2) | *NA* |
| More than once | 12 (12.9) | 8 (11.3) | 4 (18.2) | *NA* |
| Lost to follow-up at maximum setback *n (%)* | 8 (8.6) | 1 (1.4) | 7 (31.8) | **0.0001** |
| Therapeutic nonadherence after transfer *n (%)* | 20 (22.2) | 12 (16.9) | 8 (36.4) | 0.06 |
|  |  |  |  |  |
| NA: not applicable. |  |  |  |  |
