# Supplementary material for: Success of transition to adult care in patients with pediatric‐onset chronic liver disease
Source: J Pediatr Gastroenterol Nutr. 2026 Apr 20;83(1):145–54. doi: 10.1002/jpn3.70436 (PMC13342795; doi:10.1002/jpn3.70436)
Supplement: Supplementary file 3 [file JPN3-83-145-s003.docx]

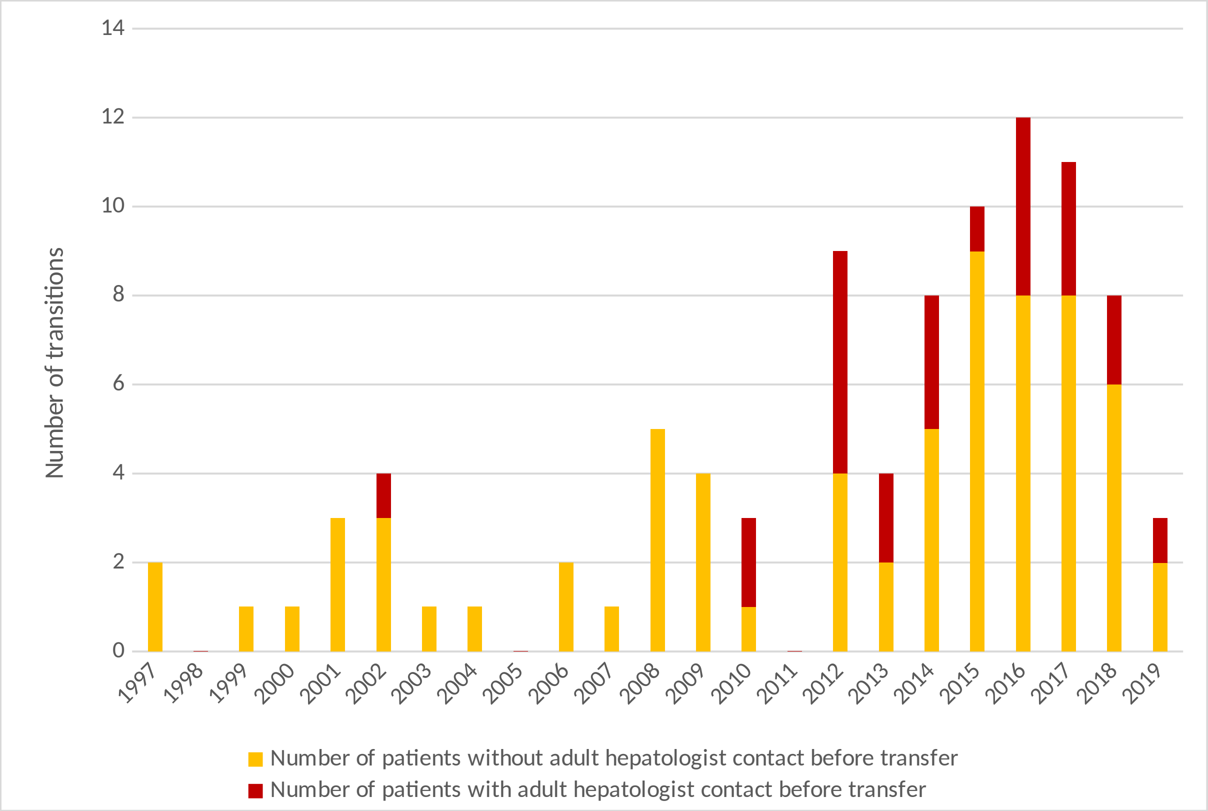

Number of transitions
14
12
10
8
6
4
2
0
1997
1998
1999
2000
2001
2002
2003
2004
2005
2006
2007
2008
2009
2010
2011
2012
2013
2014
2015
2016
2017
2018
2019
Number of patients without adult hepatologist contact before transfer
Number of patients with adult hepatologist contact before transfer
